# Supplementary material for: Small-angle X-ray microdiffraction from fibrils embedded in tissue thin sections
Source: J Appl Crystallogr. 2022 Nov 21;55(Pt 6):1562–71. doi: 10.1107/S1600576722009955 (PMC9721334; doi:10.1107/S1600576722009955)
Supplement: Supplementary file 1 [file j-55-01562-sup1.pdf]

JOURNAL OF APPLIED CRYSTALLOGRAPHY

Volume 55 (2022)

Supporting information for article:

Small-angle x-ray microdiffraction from fibrils embedded in tissue thin sections

Prakash Nepal, Abdullah Al Bashit, Lin Yang and Lee Makowski

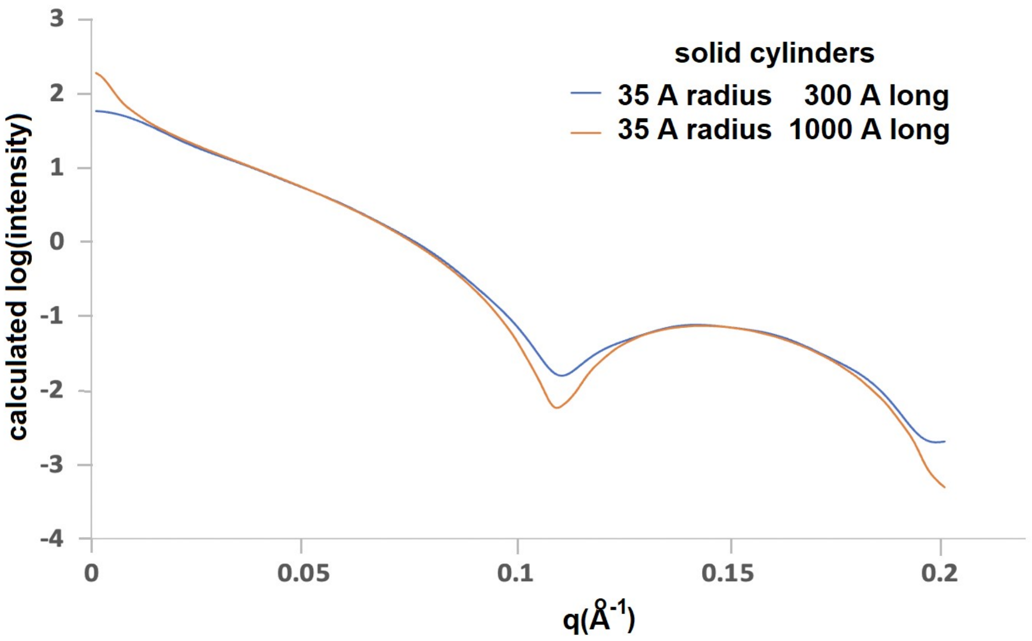

Figure S1 Calculated spherically averaged intensity for solid cylinders 35 Å in radius and 300 and 1000 Å in length. Scattering from the longer cylinder is predicted to exhibit a spike in intensity at very low scattering angles, and sharper minima in intensity.

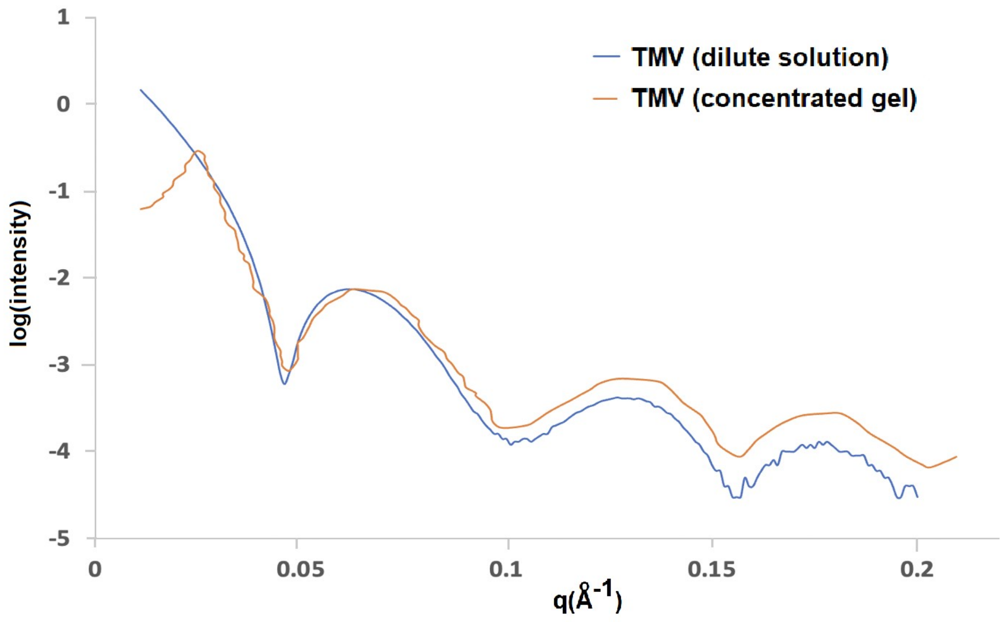

Figure S2 SAXS intensity observed from Tobacco Mosaic Virus in dilute aqueous solution and concentrated gels (after geometric correction). The intensity distributions are very similar except at small angles where interparticle interference effects dramatically alter the scattering from the concentrated gel sample.

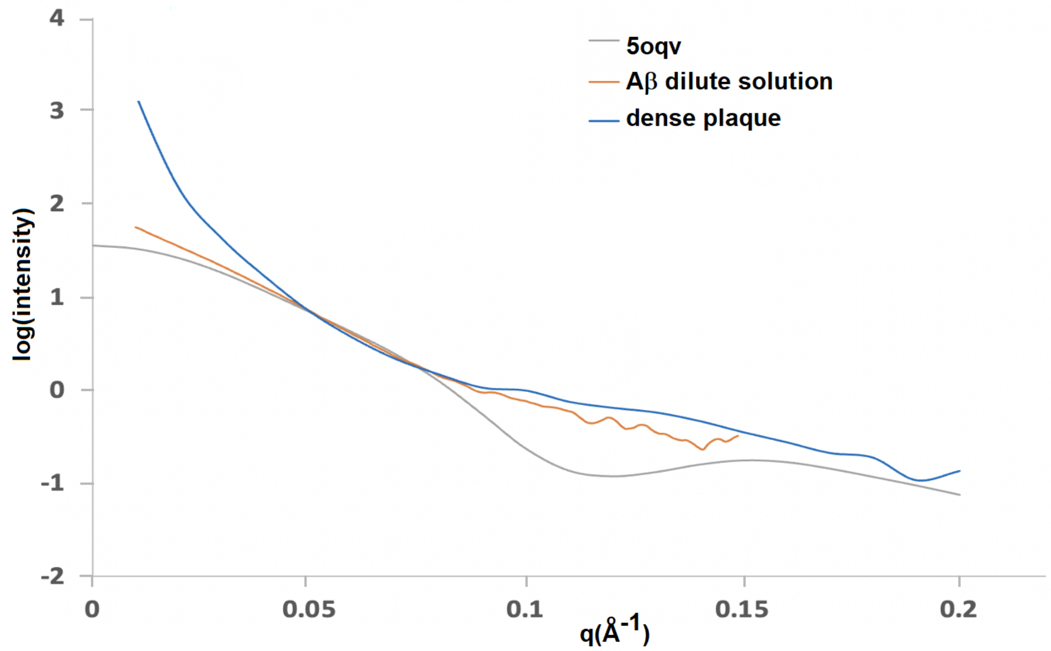

Figure S3 Comparison of SAXS data as estimated from a fibril constructed from pdb file 5oqv; solution scattering from *in vitro* assembled fibrils; microdiffraction from fibrils embedded in a histological tissue section. Note the small angle spike in scattering from the tissue section.

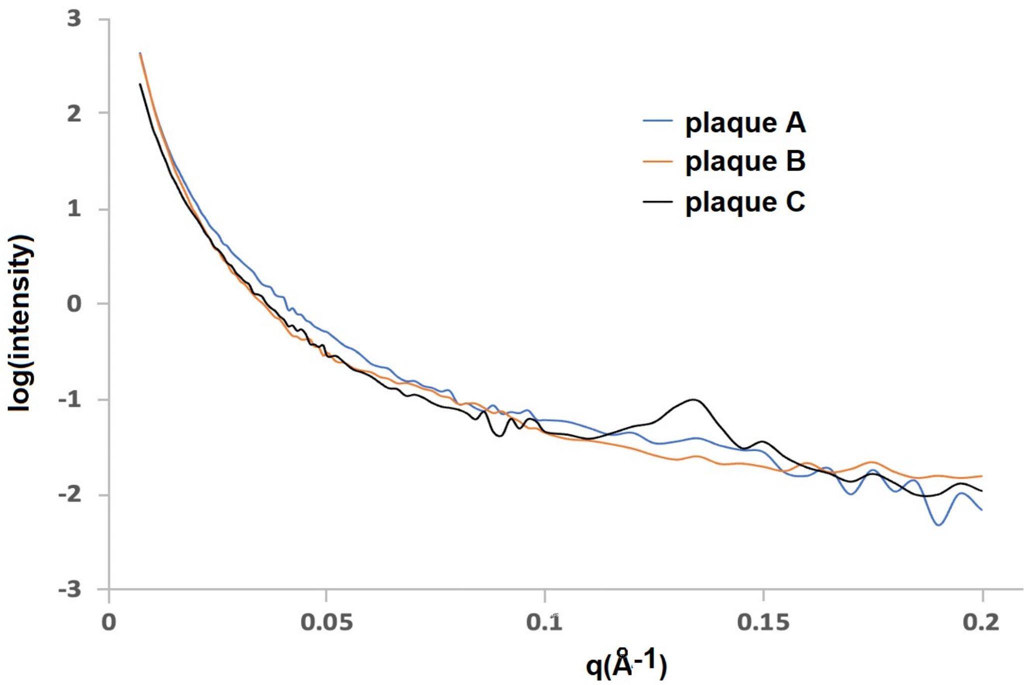

Figure S4 SAXS data from three locations in the sample exhibited in **Figure 1**. Plaque C exhibits a peak at q ~ 0.14 $Å^{-1}$ corresponding to a periodicity of ~ 50 Å as seen in the correlation function in Figure 8.
